# Hederasaponin C ameliorates chronic obstructive pulmonary disease pathogenesis by targeting TLR4 to inhibit NF-κB/MAPK signaling pathways

**DOI:** 10.1186/s13020-025-01155-5

**Published:** 2025-07-03

**Authors:** Yujie Ning, Liting Huang, Qin-Qin Wang, Lina Liu, Xinghua Ni, Xiaoyun Xie, Jingyu Liu, Qian Su, Shilin Yang, Renyikun Yuan, Hongwei Gao

**Affiliations:** 1 Engineering Research Center of Innovative Drugs for Traditional Chinese Medicine and Zhuang & Yao Medicine, Ministry of Education, Nanning, 530200 China; 2 College of Pharmacy, Guangxi University of Chinese Medicine, Nanning, 530200 China; 3 College of Pharmacy, Jiangxi University of Chinese Medicine, Nanchang, 330004 China

**Keywords:** Hederasaponin C, COPD, TLR4, Inflammation

## Abstract

**Background:**

Chronic obstructive pulmonary disease (COPD) is a complex respiratory disorder characterized by persistent respiratory symptoms and progressive airflow limitation. Long-term exposure to harmful particulates and gases causes structural abnormalities in the airways and alveoli, activating NF-κB/MAPK signaling pathways that drive chronic inflammation and tissue remodeling. Key features include an imbalance between proteolytic enzymes and inhibitors mediated by matrix metalloproteinases, and excessive mucus secretion due to mucin overexpression. These factors exacerbate airway obstruction and inflammation, contributing to disease progression. Hederasaponin C (HSC), a triterpenoid saponin with anti-inflammatory properties, shows potential in mitigating COPD-related inflammation, but its precise mechanisms require further investigation.

**Methods:**

We investigated the impact of HSC on COPD models induced by CSE + LPS using a comprehensive approach. In vitro studies included Western blotting, qRT-PCR, ELISA, and immunofluorescence to assess key proteins in NF-κB/MAPK signaling pathways, MMP9 and MMP12 expression, and mucin levels (MUC-5AC, MUC-5B). Binding affinity between HSC and TLR4 was evaluated using molecular docking, SPR analysis, and CETSA. DNA methylation at MUC-5B chr11:1243469 position was detected using an Agilent 2100 Bioanalyzer. In vivo, a COPD mouse model induced by cigarette smoke and LPS (CS + LPS) was developed, and HSC treatment effects were evaluated using H&E staining, multiplex immunofluorescence staining, Western blot, and ELISA kits.

**Results:**

HSC significantly inhibited CSE + LPS-induced inflammation by targeting TLR4 and attenuating NF-κB/MAPK signaling pathways overactivation. It also downregulated MMP9, MMP12, MUC-5AC, and MUC-5B expression and suppressed MUC-5B chr11:1243469 position DNA methylation. In vivo, HSC alleviated COPD symptoms in CS + LPS-induced mice, reducing TLR4/NF-κB/MAPK signaling pathways overactivation and smoking-associated factors.

**Conclusion:**

HSC targets TLR4, attenuates NF-κB/MAPK signaling pathways overactivation, reduces MMP9, MMP12, MUC-5AC, and MUC-5B expression, and suppresses MUC-5B chr11:1243469 position DNA methylation. These actions reduce inflammation, restore protease-antiprotease balance, and mitigate excessive mucus secretion, highlighting the promise of HSC as a viable treatment strategy for COPD management.

**Supplementary Information:**

The online version contains supplementary material available at 10.1186/s13020-025-01155-5.

## Introduction

Chronic obstructive pulmonary disease (COPD) is a complex respiratory disorder characterized by persistent respiratory symptoms and progressive airflow limitation [1]. It is primarily caused by prolonged exposure to harmful particulates and toxic gases [2]. The development of COPD involves multiple factors and mechanisms, including imbalances between proteolytic enzymes and their inhibitors, oxidative stress, and chronic inflammation [3]. Among the contributing factors, cigarette smoke (CS) is the most significant. CS alters immune responses and promotes the progression of COPD [4]. CS-induced oxidative stress activates alveolar epithelial cells, the first responders to smoke exposure, leading to the release of inflammatory mediators such as CXCL-1, CXCL-8, and TNF-α. These chemokines bind to CXC chemokine receptor 2 (CXCR-2), promoting neutrophil recruitment to the airways. Neutrophil accumulation in the lungs increases the production of proteases, reactive oxygen species (ROS), MMP-9, and MMP-12, contributing to excessive mucus production and the development of emphysema [5]. Co-exposure to CS and lipopolysaccharide (LPS) further enhances the secretion of pro-inflammatory cytokines, including TNF-α, IL-1β, and IL-6 [6]. The combination of CS and LPS is widely used as an experimental model for studying COPD pathogenesis [7].

Toll-like receptors (TLRs) are essential protein receptors involved in immune responses. Among the 11 TLRs identified in mammals, TLR4 is broadly expressed in various tissues and acts as a receptor for both endogenous and exogenous ligands, initiating immune and inflammatory responses following injury or infection [8]. Exposure to CS and LPS induces airway epithelial cells to release harmful molecules that interact with TLR4 on the cell surface [9, 10]. TLR4 activation subsequently triggers the NF-κB and MAPK signaling pathways [11]. Current treatments for COPD include long-acting antimuscarinic agents, long-acting β2 agonists, inhaled corticosteroids (ICS), and phosphodiesterase-4 (PDE4) inhibitors such as roflumilast. However, these therapies are often associated with significant side effects [12], highlighting the urgent need for novel treatment strategies to improve disease management.

Epigenetic modifications refer to reversible chemical changes to DNA and histones, including DNA methylation, histone acetylation, and the involvement of noncoding RNAs [13]. Altered epigenetic patterns have been associated with COPD progression, reflecting the combined influence of environmental exposures (e.g., smoking and air pollution) and genetic predisposition [14]. Both COPD and lung cancer share smoking as a major risk factor. Mechanisms such as immune dysfunction, lung microbiota imbalance and infection, epithelial-mesenchymal transition, DNA damage, and tumor angiogenesis contribute to the link between COPD and lung cancer development [15]. Overexpression of airway mucins has been reported in chronic inflammatory lung diseases, including cystic fibrosis, asthma, idiopathic pulmonary fibrosis, and COPD [16], many of which are associated with increased lung cancer risk [17]. Studies have shown that demethylation of the MUC-5B promoter region leads to elevated gene expression. Epigenetic silencing of MUC-5B may serve as a mechanism by which cancer cells maintain an undifferentiated state or by which normal cells delay differentiation [18]. Therefore, targeting the epigenetic regulation of MUC-5B may represent a potential therapeutic strategy for COPD [19].

Hederasaponin C (HSC), the major bioactive component of *Pulsatilla chinensis* (Bunge) Regel, is a triterpene saponin commonly used in traditional Chinese medicine. HSC exhibits strong anti-inflammatory properties and has demonstrated therapeutic effects in colitis [20],acute lung injury [21], and acute kidney injury [22]. However, its potential efficacy and mechanisms in COPD treatment have not been explored. This study aims to evaluate the therapeutic effects of HSC in COPD and investigate the underlying mechanisms using both in vivo and in vitro experimental approaches.

## Materials and methods

### Reagents

**Fig. 1:**
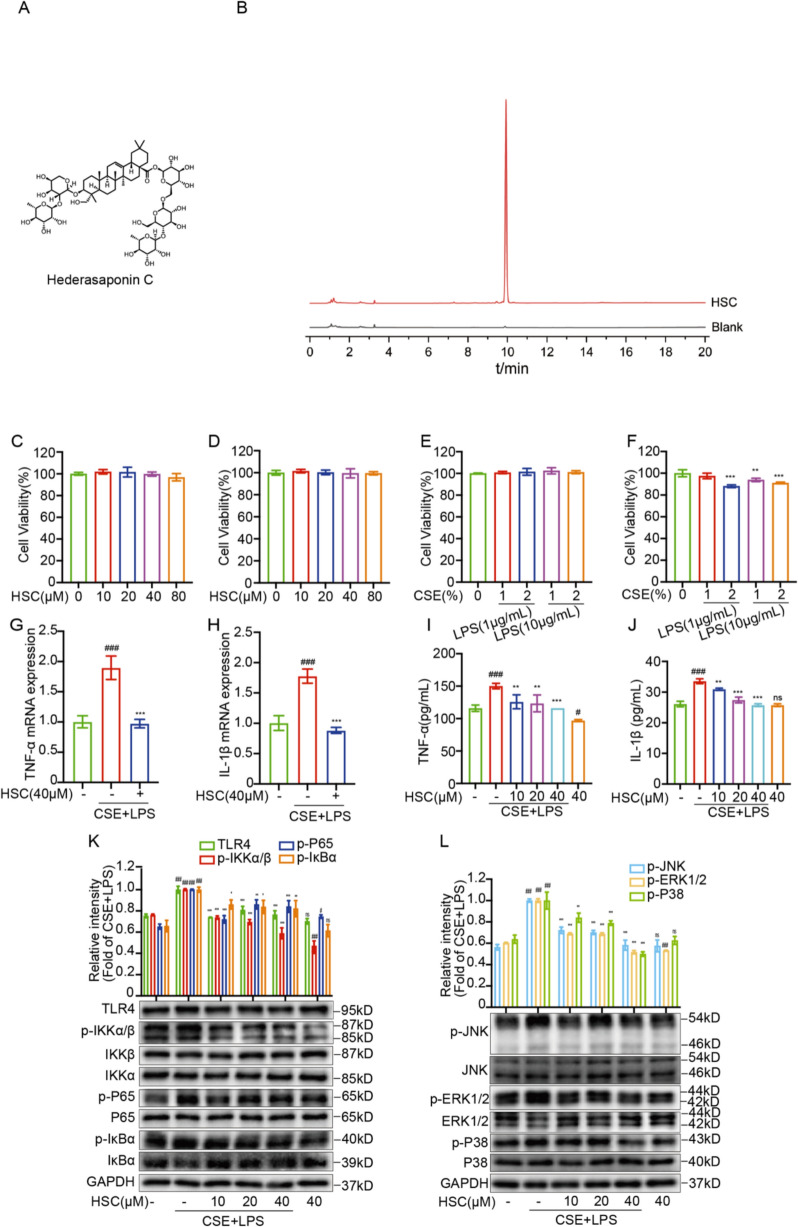
HSC suppresses TLR4/NF-κB/MAPK signaling and pro-inflammatory cytokines expression in CSE + LPS-induced A549 cells. **A** The chemical structure of HSC. **B** The HPLC chromatograms of HSC. **C**, **D** The MTT assay was used to evaluate the cytotoxicity of HSC (0, 10, 20, 40, 80 μM) for 24 h and 48 h. **E**, **F** The MTT assay was used to evaluate the cytotoxicity of CSE + LPS (1%CSE + 1 μg/mL LPS, 2%CSE + 1 μg/mL LPS, 1%CSE + 10 μg/mL LPS, 2%CSE + 10 μg/mL LPS) for 24 h and 48 h. **G**, **H** The mRNA levels of TNF-ɑ and IL-1β were determined by RT-qPCR. **I**, **J** The expression levels of TNF-ɑ and IL-1β were determined by ELISA kits. **K**, **L** The expression of TLR4, phosphorylated P65, IKKα/β, IκBα, JNK, ERK1/2, and P38 were detected by Western blotting. ^#^*P* < 0.05, ^###^*P* < 0.001 vs control group; ^*^P < 0.01, ^**^P < 0.01, ^***^P < 0.001 vs CSE + LPS group

Hederasaponin C (HSC) was self-extracted in the laboratory, with a purity greater than 98% (The HPLC chromatogram of HSC is shown in Fig. 1B, The NMR spectrum of HSC is shown in Supplementary file 1.). LPS (derived from Escherichia coli O111:B4), TAK242, and MTT were sourced from Sigma-Aldrich, which is based in St. Louis, Missouri, USA. Zhenlong cigarettes (Tar content:1.1 mg, Nicotine content in smoke: 1.1 mg, the amount of carbon monoxide in flue gas:12 mg) was purchased from China Tobacco Guangxi Industry Co., Ltd. Nucleic acid extraction reagent kit (magnetic bead method) was purchased from GEFINE (Changzhou, China). Acegen Targeted Methyl Panel Kit (Cat. No. AG0508) was purchased from Acegen. The EZ DNA Methylation-Gold Kit was acquired from ZYMO Research, a company based in Irvine, CA, USA. The primary antibodies against GAPDH (#5174S), IKKα (#2682S), IKKβ (#8943S), phospho-IKKα/β (#2697S), IκBα (#4814T), phospho-IκBα (#2859S), phospho-p65 (#3033S), phospho-JNK (#4668S), JNK (#9252S), ERK1/2 (#4695T), phospho-ERK1/2 (#4370S), p38 MAPK (#9212S), and phospho-p38 MAPK (#4511S) were provided by Cell Signaling Technology (Beverly, MA, USA).MMP9 (10375-2-AP) and MMP12 (22989-1-AP) were provided by Proteintech (Wuhan, China). TLR4 (48–2300) was sourced from Invitrogen, a company located in Carlsbad, California, United States.P65 (ET1603-12) was purchased from HUABIO (Hangzhou, China). MUC-5AC (YN0880) and MUC-5B (YN0881) were purchased from Immunoway (Suzhou, China). The Goat Anti-Rabbit IgG, HRP-conjugated Antibody (GB23303) was provided by Wuhan servicebio technology CO., Ltd., headquartered in Wuhan, China. Goat Anti-Rabbit IgG AF488 (#M21012) was purchased from Abmart (Shanghai, China). The ELISA kits for mouse TNF-α (F2132-A) and IL-1β (F2040-A) were provided by Shanghai Sinovac Trading Co., Ltd., headquartered in Shanghai, China. The ELISA kits for human TNF-α (S0C3024) and IL-1β (S0C3013) were provided by STARTER, headquartered in Hangzhou, China. The DAPI staining reagent (G1012) was provided by Wuhan servicebio technology CO., Ltd., headquartered in Wuhan, China.

### Cell culture

A549 and HEK293T cells were procured from ATCC (USA) and maintained in RPMI 1640 and DMEM media, respectively. These media were enriched with 10% fetal bovine serum and 1% penicillin/streptomycin, all provided by Gibco (USA). Culturing of the cells was performed in a humidified incubator at 37 °C with an atmosphere containing 5% CO₂.

### Preparation of cigarette smoke extract (CSE)

In vitro experiment, the same Zhenlong cigarettes used in the in vivo experiment was employed to prepare the CSE. The procedure followed the methods described in the literature [23, 24] with slight modifications: A cigarette was connected to one end of a large Bausch tube containing 20 mL of serum-free RPMI 1640 culture medium, while the other end was connected to a vacuum pump. After lighting the cigarette, the vacuum pump was activated to simulate human smoking conditions. Suction continued until 2.0–3.0 mm from the cigarette butt, and two cigarettes were extracted consecutively. Upon completion of the suction step, the solution was thoroughly combined and subsequently transferred to a 50 mL sterile centrifuge tube, ensuring complete dissolution of CS in the absorption liquid. The pH of the extract was adjusted to 7.40 ± 0.02 with NaOH, and the solution was sterilized and particle-free by passing it through a 0.22 µm microporous filter. For standardization, the OD value at 320 nm was measured using a microplate reader (BioTek, Vermont, USA), confirming an OD value within 0.80 ± 0.05. This established the 100% CSE stock solution, which was stored at − 80 °C and diluted to the desired concentration when needed.

### Cell viability assay

A549 cells were pre-incubated with HSC (10, 20, 40, 80 μM), or with combinations of CSE and LPS (1% CSE + 1 μg/mL LPS, 2% CSE + 1 μg/mL LPS, 1% CSE + 10 μg/mL LPS, 2% CSE + 10 μg/mL LPS). Additionally, cells were pre-treated with HSC in combination with the TLR4 inhibitor TAK-242 (10 μM) before being incubated with CSE + LPS for 24 h and 48 h, respectively. The cell cultures were exposed to MTT solution and incubated for a duration of 4 h. Next, the absorbance at 490 nm was assessed with a multilabel microplate reader produced by BioTek (Vermont, USA).

### DNA methylation analysis

For the MUC-5B promoter region, BS-PCR primers were designed and synthesized. The MUC-5B primer sequence information are provided in the Supplementary file 2. Cell samples were processed for DNA extraction using the magnetic bead method with a reagent kit, in line with the manufacturer’s guidelines. After verifying the quality of the extracted DNA, targeted bisulfite sequencing was conducted following previously established methods [25–28], gene-specific DNA methylation levels were assessed using bisulfite sequencing based on next-generation sequencing technology. Targeted bisulfite sequencing libraries were built using the Acegen Targeted Methyl Panel Kit following the instructions provided by the manufacturer. Genomic DNA (500 ng) underwent treatment with the ZYMO EZ DNA Methylation-Gold Kit, and the resultant product served as a template for PCR amplification, performing 10 cycles. For each sample, the amplified product underwent end-repair, 3’-dA-tailing, and ligation of a 5-methylcytosine-modified adapter. The constructed library was analyzed with an Agilent 2100 Bioanalyzer and subsequently sequenced on an Illumina platform using a 150 × 2 paired-end sequencing approach. The methylation level at each CG site within the target sequence was determined as follows: Methylation level = number of reads supporting methylation/(number of reads supporting methylation + number of reads supporting non-methylation).

### Quantitative real-time PCR (qRT-PCR) assay

A549 cells were subjected to pre-incubated with or without HSC (40 μM), and then incubated with CSE + LPS for either 3 h or 6 h. Total RNA extraction was performed, and 1 μg of the extracted RNA was used for qRT-PCR analysis with SYBR Green reagent. The sequences of the oligonucleotide primers for TNF-α, IL-1β, MMP9, MMP12, MUC-5AC, MUC-5B, and GAPDH are detailed in Supplementary file 3.

### Western blotting analysis

A549 cells were pre-incubated with HSC (10, 20, 40 μM) for 4 h, and then incubation with CSE + LPS for 24 h or 48 h. Cell lysis was performed using RIPA buffer supplemented with 1% cocktail and 1% PMSF. After centrifugation, the supernatant from the lysate was collected. Protein concentrations were determined using a BCA protein assay kit. The proteins were separated by SDS-PAGE, transferred to PVDF membranes, and blocked with 5% nonfat milk for 2 h. The membranes were then incubated with primary antibodies overnight at 4 °C, followed by incubation with a secondary antibody (diluted 1:5000) at room temperature for 2 h. Finally, protein bands were detected using the ChemiDoc™ MP Imaging System from Bio-Rad (Hercules, CA, USA).

### Immunofluorescence assay

A549 cells were subjected to pre-incubated with or without HSC (40 μM), and then incubated with CSE + LPS for either 24 h or 48 h. The cells were first washed twice with PBS, then fixed in 4% paraformaldehyde at room temperature for 30 min. Next, they were treated with 0.1% Triton X-100 for 15 min to facilitate permeabilization. Non-specific binding sites were blocked by incubating the samples with a 5% bovine serum albumin solution for 30 min. Primary antibodies against P65, MMP9, MUC-5AC, and MUC-5B were applied, and the samples were incubated overnight at 4 °C. The samples were then incubated with Goat Anti-Rabbit IgG AF488 for 2 h at room temperature. Hoechst 33342 staining was performed for 15 min. Fluorescent images were ultimately captured using a Cytation 5 instrument (BioTek, USA).

### Cell thermal shift assay (CETSA)

HEK293T cells were disrupted using RIPA buffer supplemented with 1% cocktail and 1% PMSF. The resulting cell lysate was divided into two fractions: one fraction was treated with DMSO, whereas the other fraction received a 40 μM HSC treatment. Following 1 h incubation on ice, the samples were centrifuged at 15,000 rpm for 25 min at 4 °C. The supernatant was harvested and partitioned into seven equal aliquots, which were then subjected to heat treatment at varying temperatures (45, 49, 53, 57, 61, 65, and 69 °C) for 3 min each. Following heat treatment, the samples were cooled to room temperature for 30 s before proceeding with Western blot analysis.

### Molecular docking

Acquire the 3D structural data of protein TLR4 (PDB ID: 3FXI) from the PDB repository. The protein structures for docking were examined using PyMOL 2.3.0. The ligand small molecule was HSC (CAS ID: 14216-03-6), and its 3D structure file was downloaded from the PubChem database. The molecular structure was subsequently optimized with the MMFF94 force field implemented in OpenBabel3.1.1 to obtain the lowest energy state. AutoDock Tools1.5.6 was used to add hydrogen atoms to the protein and the small molecule, define the torsion bonds, and save the files in pdbqt format. Using the Grid panel, the docking parameters for the protein were set to employ a semi-flexible docking approach with an exhaustiveness of 25. The docking simulations were conducted using the Lamarckian genetic algorithm, yielding 20 binding poses in the final results. AutoDock Vina 1.2.0 software was employed to carry out the molecular docking and compute the binding free energy.

### Surface plasmon resonance (SPR) analysis

The interaction between HSC and TLR4 was assessed using a Biacore X100 system (Cytiva, USA). Human recombinant TLR4 protein was immobilized on the CM5 chip’s flow channels. HSC solutions, prepared in HBS-EP buffer (Cytiva, USA), were introduced into the system at various concentrations and a flow rate of 20 μL/min. Binding kinetics and kinetic parameters were analyzed with the Kinetic Analysis Wizard software. For the analysis of data, the x-axis depicted time, whereas the y-axis showed response units.

### CS combined with LPS to induce COPD mice

The animal experiment was approved by Guangxi University of Chinese Medicine Institutional Welfare and Ethical Committee (Approval No. DW20250113-SY08). Male C57BL/6 J mice in good health (SPF grade, aged 6–8 weeks, with a weight of 18–22 g) were acquired from Guangdong Wei Tong Li Hua Experimental Animal Technology Co., Ltd. (Animal License No. SCXK (Yue) 2022-0063, Guangdong Province, China). All animals were maintained in a SPF environment, with the ambient temperature set at 25 °C and relative humidity at 50%. They had unrestricted access to both food and water. After a 3 days adaptation with feeding, all groups except the control group were subjected to LPS nasal drops combined with CS exposure to establish COPD mouse models (each group has 10 mice). On the first day of modeling, the model group, HSC (4 mg/kg and 8 mg/kg) groups, and the Dexamethasone (DEX,5 mg/kg) group were administered 7.5 μg/50μL LPS nasal drops, with 25 μL per mouse. On the second day, CS exposure modeling commenced. Each session involved exposing the animals to 10 cigarettes for 30 min. Specifically, cigarettes were lit and allowed to burn completely, which took approximately 10 min. The remaining smoke was then used for an additional 20 min. The modeling process lasted for 5 weeks, and HSC administration commenced 1 week after the initiation of modeling. Continuous aerosol inhalation of HSC was administered for 4 weeks concurrently with the ongoing modeling. DEX (5 mg/kg, i.p.) was administered once weekly. LPS nasal drops were administered for 10 consecutive days, and CS exposure modeling was not performed on the days of nasal drop administration. On the 36th day, venous blood specimens were acquired, and the mice were euthanized through cervical dislocation. Whole blood was analyzed with an automated hematology analyzer (Mindray, Shenzhen, China). Concentrations of inflammatory mediators in bronchoalveolar lavage fluid (BALF) and lung tissue samples were analyzed using ELISA kits. Lung tissue samples underwent histological analysis and Western blotting for additional evaluation.

### Enzyme-linked immunosorbent assay (ELISA)

The cell supernatant and BALF were underwent centrifugation at 4 °C for 15 min at 1500 rpm, after which the supernatant was retrieved to serve as the cell supernatant and BALF samples. For lung tissue processing, homogenization was performed using a KZ-III-F tissue grinder (Servicebio®, China), followed by centrifugation under the same conditions (4 °C, 1500 rpm, 15 min). The resultant supernatant was then collected and preserved at − 80 °C. The levels of inflammatory markers, specifically TNF-α and IL-1β, were assessed in cell supernatant, BALF, and lung tissue samples through ELISA assays, adhering to the manufacturer’s guidelines.

### Histopathological analysis

Hematoxylin and eosin (H&E) staining was employed to evaluate histopathological alterations. Tissue samples from the lung, heart, liver, spleen, kidney, and brain were fixed in 4% paraformaldehyde solution for 24 h, followed by a second fixation in fresh 4% paraformaldehyde. Following this, H&E staining was conducted. Additionally, multiplex immunofluorescence staining was conducted on sections of lung tissue for TLR4, MMP9, MMP12, MUC-5AC, and MUC-5B.

### Data analysis

Data are expressed as the mean ± standard deviation (SD), with experiments performed in triplicate or more. Statistical analysis was conducted using one-way ANOVA in Prism 10.0 (GraphPad, USA), with statistical significance set at *P* < 0.05.

## Result

### HSC suppresses TLR4/NF-κB/MAPK signaling and pro-inflammatory cytokines expression in CSE + LPS-induced A549 cells

Figure 1A shows the chemical structure of HSC. The results demonstrated that, within 24 h, pretreatment with different concentrations of HSC and different concentrations of CSE + LPS had no significant impact on the survival of A549 cells (Fig. 1C, E). Exposure to different concentrations of HSC for 48 h did not markedly influence the viability of A549 cells with respect to cytotoxic effects (Fig. 1D). Conversely, when A549 cells were subjected to different concentrations of CSE + LPS for the 48 h, only the combination of 1% CSE and 1 μg/mL LPS did not affect cell viability. Other concentration levels exhibited marked cytotoxicity. Consequently, subsequent experiments utilized 1% CSE + 1 μg/mL LPS to induce A549 cells as an in vitro experimental model of COPD (Fig. 1F).

Western blot analysis revealed that HSC significantly inhibited the activation of the TLR4/NF-κB/MAPK signaling pathways. The expression of TLR4, phosphorylated P65, IKKα/β, IκBα, JNK, ERK1/2, and P38 proteins were upregulated in A549 cells induced by CSE + LPS, while HSC could inhibit these phosphorylation proteins expression (Fig. 1K, L). Activation of TLR4 is known to trigger the downstream NF-κB/MAPK signaling pathways, resulting in the initiation of inflammatory reactions [29]. To further investigate the influence of HSC on pro-inflammatory cytokine release, RT-qPCR and ELISA were employed. In CSE + LPS-induced A549 cells, there was a significant upregulation in the expression levels of TNF-α and IL-1β. Notably, HSC treatment effectively inhibited this increase (Fig. 1G–J).

### HSC reduces MUC-5AC and MUC-5B expression and reverses MUC-5B hypomethylation in CSE + LPS-induced A549 cells

**Fig. 2:**
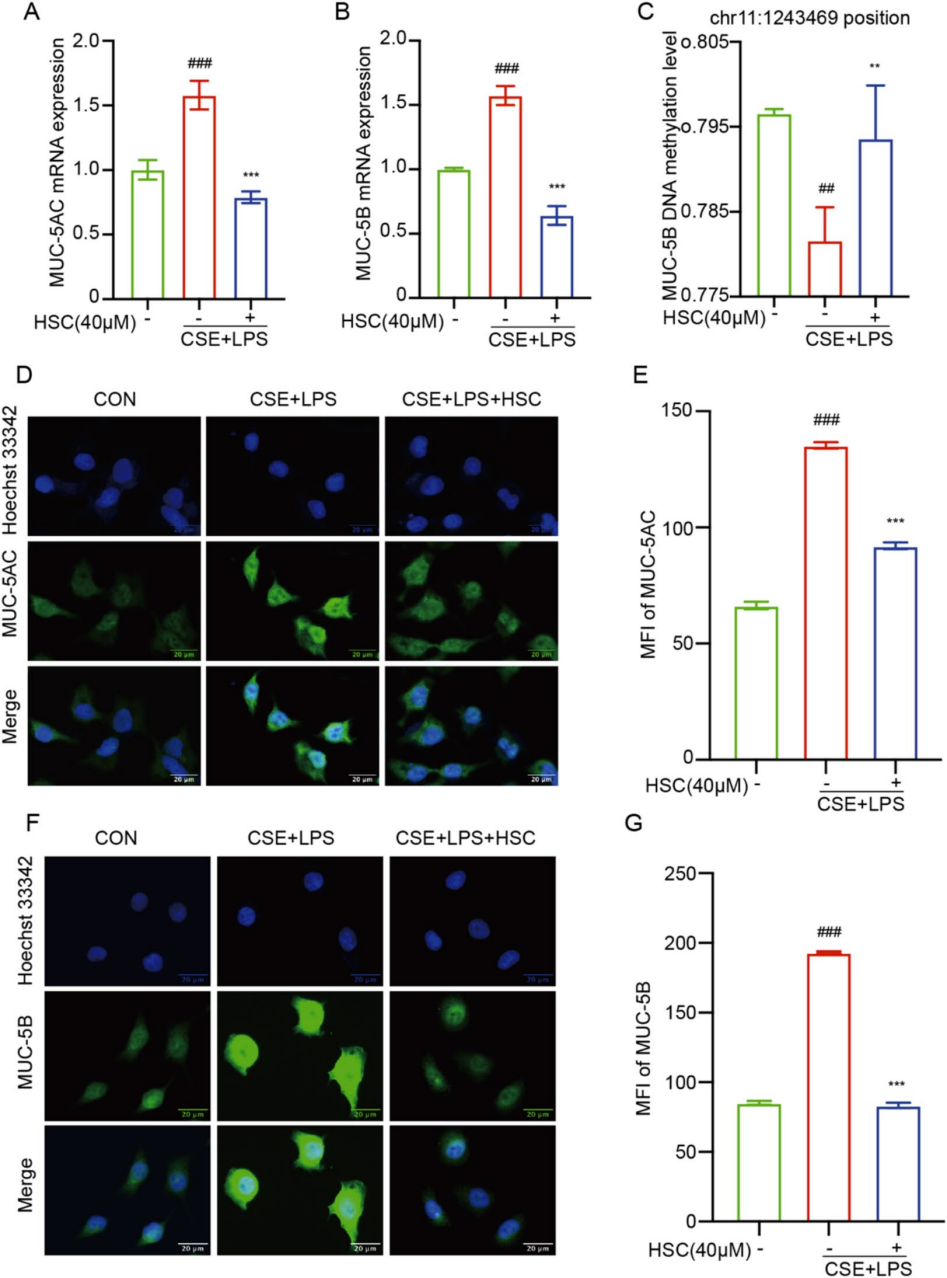
HSC reduces MUC-5AC and MUC-5B expression and reverses MUC-5B hypomethylation in CSE + LPS-induced A549 cells. **A**, **B** The mRNA levels of MUC-5AC and MUC-5B were determined by RT-qPCR. **C** The Ailent 2100 Bioanalyzer was utilized to detect the DNA methylation level of the MUC-5B chr11:1243469 position. **D**–**G** The immunofluorescence of the expression of MUC-5AC and MUC-5B proteins and the statistical chart of MFI (Scale bar = 20 μm). ^##^*P* < 0.01, ^###^*P* < 0.001 vs control group; ^**^P < 0.01, ^***^P < 0.001 vs CSE + LPS group

While mucins MUC-5AC and MUC-5B are the principal secreted mucins found in the airways of healthy individuals, their levels are substantially elevated in patients with moderate COPD compared to both non-smokers and smokers without airway obstruction [30]. In this current study, HSC was found to markedly decrease the mRNA levels of MUC-5AC and MUC-5B in CSE + LPS treatment A549 cells (Fig. 2A, B). MUC-5B is a major component of airway secretions, plays a critical role in COPD pathogenesis, where its abnormal overexpression leads to excessive mucus production, further exacerbating airway obstruction. Results demonstrated that CSE + LPS treatment reduced the DNA methylation level of MUC-5B at the chr11:1243469 position, while HSC treatment effectively reversed this reduction, restoring DNA methylation levels (Fig. 2C). Immunofluorescence staining additionally confirmed that HSC suppressed the protein expression levels of both MUC-5AC and MUC-5B (Fig. 2D–G). These findings underscore the potential of HSC to regulate mucin expression and mitigate mucus hypersecretion in COPD.

### HSC attenuates MMP9 and MMP12 expression and tissue remodeling in CSE + LPS-induced A549 cells

**Fig. 3:**
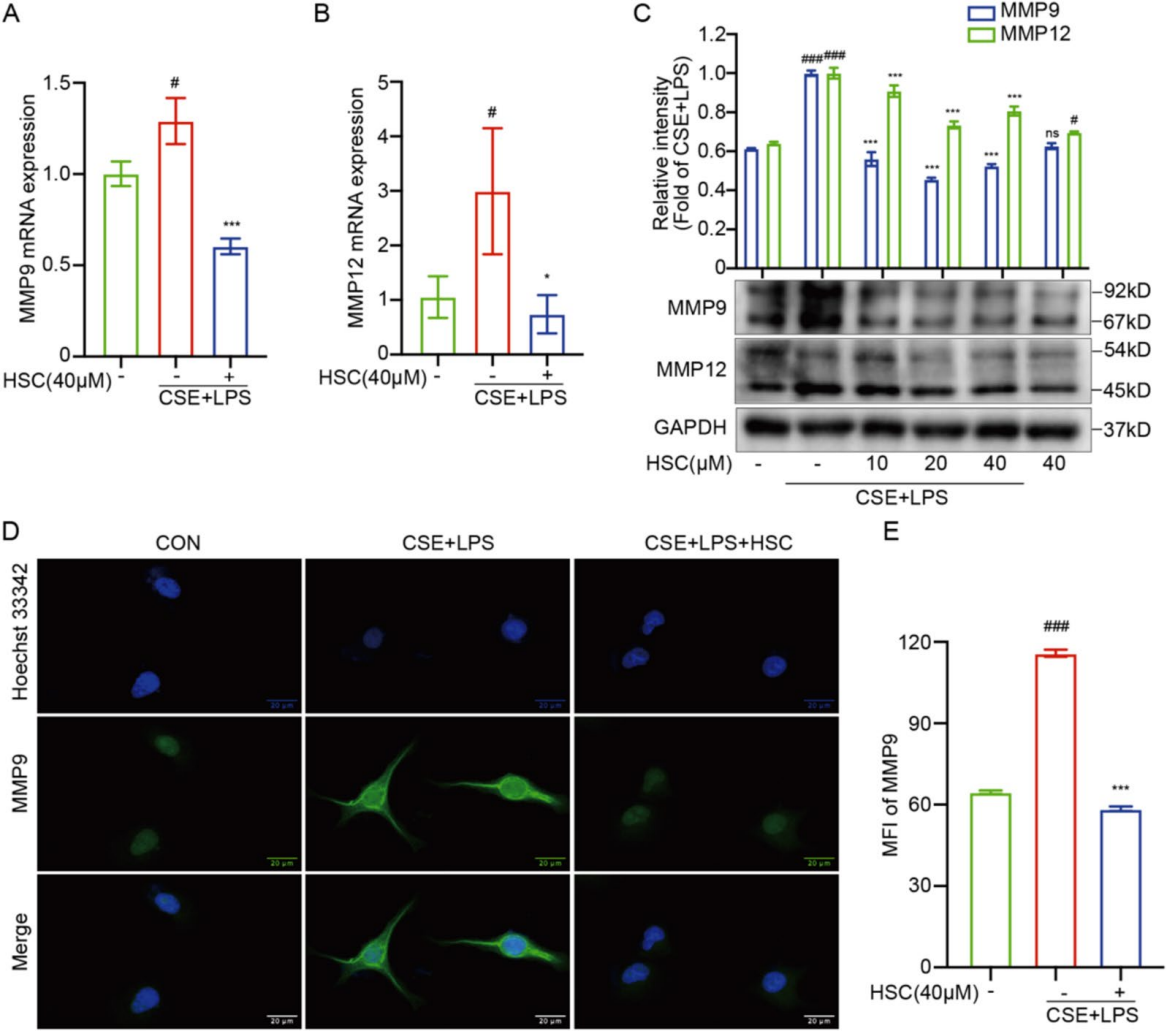
HSC attenuates MMP9 and MMP12 expression and tissue remodeling in CSE + LPS-induced A549 cells. **A**, **B** The mRNA levels of MMP9 and MMP12 were determined by RT-qPCR. **C** The expression levels of MMP9 and MMP12 proteins were assessed using Western blotting. **D**, **E** The immunofluorescence of MMP9 protein expression and the statistical chart of MFI (Scale bar = 20 μm). ^#^*P* < 0.05, ^###^*P* < 0.001 vs control group; ^∗^*P* < 0.05, ^**^P < 0.01, ^***^P < 0.001 vs CSE + LPS group

Studies have demonstrated that MMP9 and MMP12 are critical mediators participating in the inflammatory response and tissue remodeling implicated in the development and evolution of COPD [31, 32]. These proteases contribute to the breakdown of extracellular matrix components, leading to airway remodeling and emphysema, key pathological features of COPD. Our findings from qRT-PCR analysis indicated that HSC substantially reduced the mRNA levels of MMP9 and MMP12 in A549 cells exposed to CSE + LPS (Fig. 3A, B). In line with these findings, Our Western blot analysis revealed that HSC significantly reduced the protein expression levels of MMP9 and MMP12 in A549 cells following exposure to CSE + LPS (Fig. 3C), further supporting its regulatory effect on these proteases at the translational level. Moreover, immunofluorescence staining further validated that HSC markedly reduced the MMP9 protein expression in A549 cells that were exposed to CSE + LPS (Fig. 3D, E). These findings demonstrate that HSC mitigates the protease-antiprotease imbalance, a hallmark of COPD pathology.

### HSC targets TLR4 to suppress NF-κB/MAPK signaling and inflammation in CSE + LPS-induced A549 cells

**Fig. 4:**
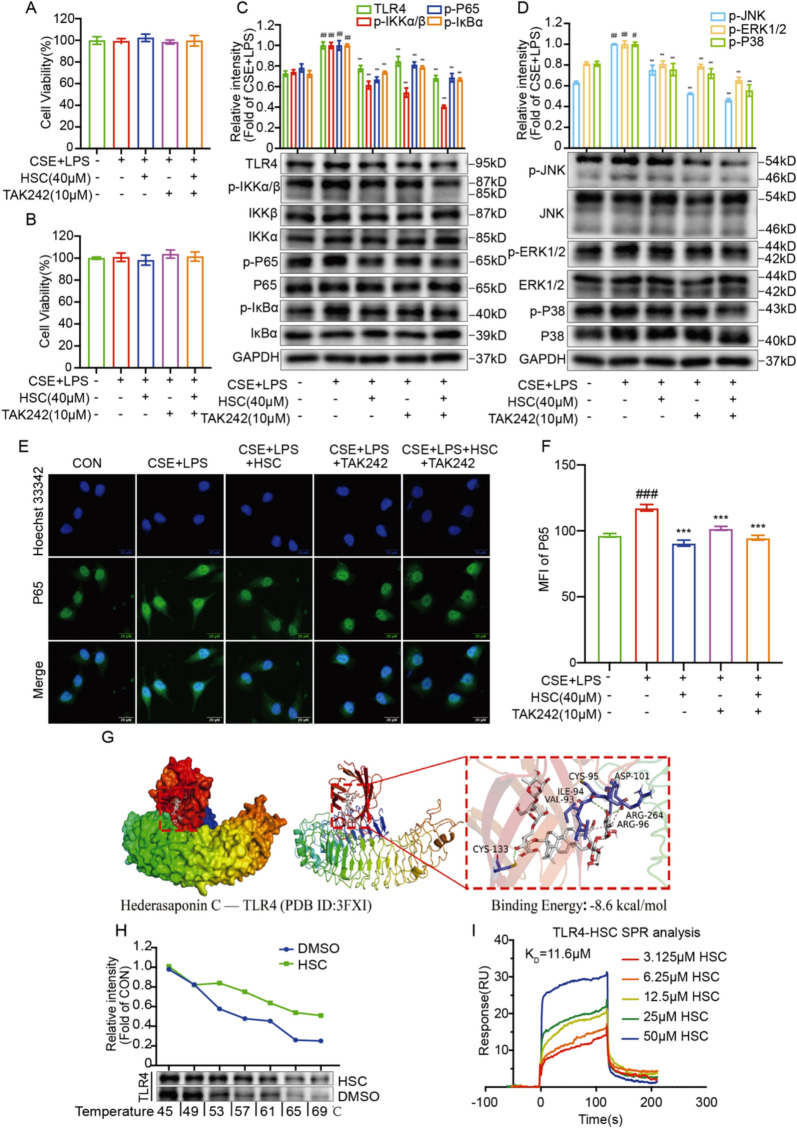
HSC targets TLR4 to suppress NF-κB/MAPK signaling and inflammation in CSE + LPS-induced A549 cells. **A**, **B** HSC and TLR4 inhibitor TAK242 (10 μM) were pretreated and incubated with CSE + LPS for 24 h and 48 h of treatment, MTT assay was employed to determine the cytotoxicity. **C**, **D** The expression of TLR4, phosphorylated P65, IKKα/β, IκBα, JNK, ERK1/2, and P38 were evaluated via Western blotting. **E**, **F** The immunofluorescence of P65 translocation and the statistical chart of MFI (Scale bar = 20 μm). **H** To investigate the stability of the TLR4 protein, HEK293T cells were exposed to 40 μM HSC and then incubated at temperatures between 45 and 69 °C. **G** Molecular docking analysis between TLR4 and HSC. **I** SPR analysis demonstrating the binding of HSC to TLR4 at different concentrations. ^#^*P* < 0.05 and ^###^*P* < 0.001 vs control group; ^***^P < 0.001 vs CSE + LPS group

Emerging evidence demonstrates that TLR4 acts as an upstream regulator capable of activating the downstream NF-κB and MAPK signaling pathways [11]. The results showed that within 24 h or 48 h, pretreated a combination of HSC and the TLR4 inhibitor TAK242 (10 μM), followed by incubation with CSE + LPS, these treatments did not significantly affect the survival of A549 cells (Fig. 4A, B). Western blot analysis showed that combination the TLR4 inhibitor TAK242 and HSC effectively inhibited the protein expression of TLR4, phosphorylated P65, IKKα/β, IκBα, JNK, ERK1/2, and P38 in A549 cells induced by CSE + LPS (Fig. 4C, D). Immunofluorescence staining further confirmed that the combined treatment of TAK242 and HSC collaboratively inhibited P65 nuclear translocation (Fig. 4E, F). Molecular docking analysis identified the specific binding site of HSC to TLR4, with a predicted binding energy of −8.6 kcal/mol (Fig. 4G), which was further validated using the Biacore X100, which showed a dissociation constant (*K*_D_) of 11.6 μM (Fig. 4I). Furthermore, CETSA results demonstrated that HSC stabilized TLR4 by protecting it from thermal degradation (Fig. 4H). Overall, these findings demonstrate that HSC targets TLR4 directly, which in turn inhibits NF-κB/MAPK signaling pathways overactivation and alleviates cellular inflammation caused by CSE + LPS in A549 cells.

### HSC alleviates COPD-related inflammation and airway remodeling via TLR4/NF-κB/MAPK pathway inhibition

**Fig. 5:**
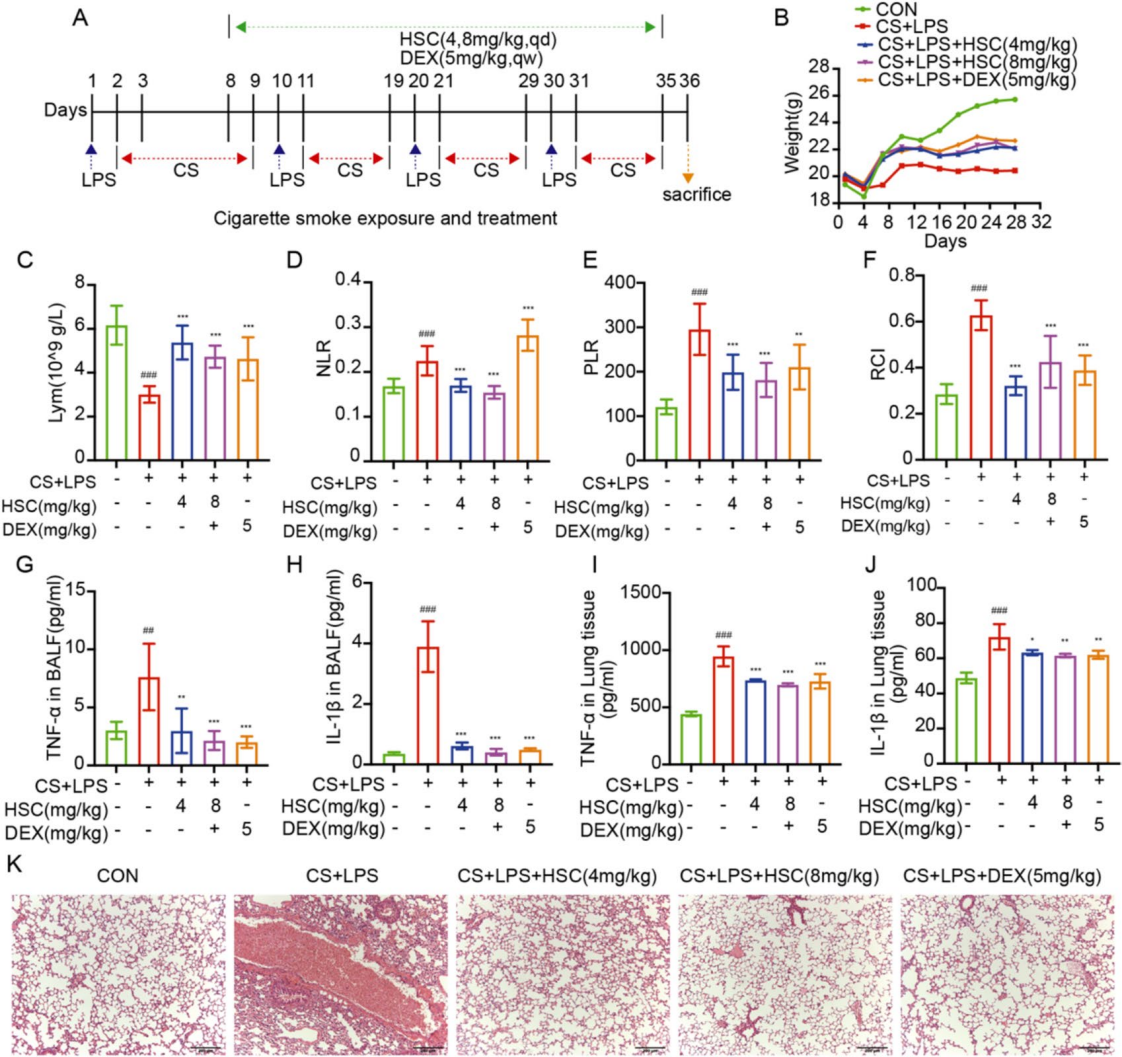
HSC alleviates COPD-related inflammation and airway remodeling via TLR4/NF-κB/MAPK pathway inhibition. **A** was the schematic diagram of the drug administration regimen for mice. **B** Weight changes in COPD mice during drug administration(n = 8). **C**–**F** Lymphocyte counts, NLR, PLR, RCI in the blood, analyzed with an automated hematology analyzer(n = 8). **G**–**J** TNF-α and IL-1β secretion were measured in BALF and lung tissues using ELISA kits(n = 4). **K** H&E staining was performed on mice lung tissue sections (Scale bar = 200 μm). All the experiments were performed three times independently. ^##^*P* < 0.01, ^###^*P* < 0.001 vs control group; ^∗^*P* < 0.05, ^∗∗^*P* < 0.01, ^***^P < 0.001 vs CS + LPS group

Evidence indicates that exposure to CS and LPS infusion exacerbate lung inflammation, closely mimicking the pathophysiology of human COPD [12]. Consequently, we implemented a combination of CS exposure and intranasal LPS administration to establish a mouse model of COPD. The neutrophil–lymphocyte ratio (NLR) and platelet-lymphocyte ratio (PLR) serve as readily existing biomarkers of systemic inflammation, often associated with disease severity, exacerbation frequency, and increased hospitalization rates. Elevated NLR levels, typically resulting from reduced lymphocyte counts, have been correlated with elevated mortality rates in elderly individuals with severe COPD [33, 34]. The red blood cell index (RCI) is correlated with platelet count (PLT), red blood cell count (RBC), hemoglobin concentration (Hb), and lymphocyte count (Lym). The equation for calculating RCI is: (RBC × Hb)/(Lym × PLT). RCI exhibits an inverse relationship with lung function and serves as a novel biomarker that provides a more accurate assessment of lung function and COPD severity compared to the NLR and PLR [35]. Figure 5A illustrates the schematic diagram of the drug administration regimen for mice. Following CS + LPS-induced injury, the body weight of mice significantly decreased. However, HSC treatment significantly increased the body weight of COPD mice (Fig. 5B).

HSC effectively increased lymphocyte counts and reduced NLR, PLR, and RCI in COPD mice (Fig. 5C–F). In contrast, the DEX group failed to reduce the NLR, possibly due to glucocorticoid resistance linked to neutrophilic airway inflammation. This mechanism may be related to oxidative stress-induced histone deacetylase 2 (HDAC2) expression and activity reduction [36]. Notably, HSC significantly diminished the levels of inflammatory cytokines, such as TNF-α and IL-1β, in BALF and lung tissue (Fig. 5G–J).

**Fig. 6:**
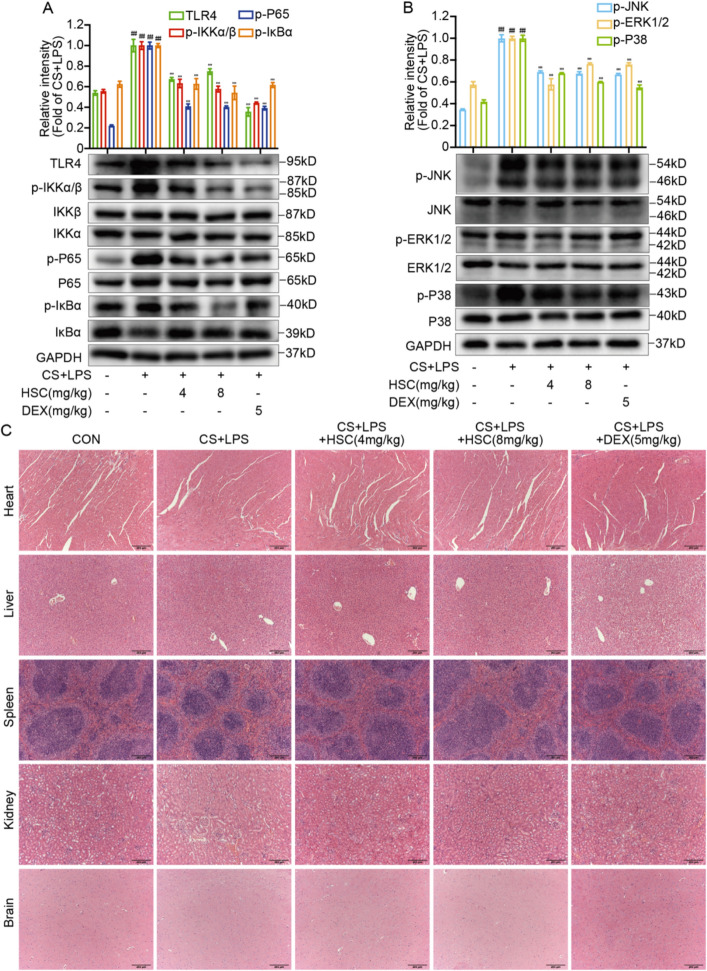
HSC alleviates COPD-related inflammation and airway remodeling via TLR4/NF-κB/MAPK pathway inhibition. **A**, **B** The expression of TLR4, phosphorylated P65, IKKα/β, IκBα, JNK, ERK1/2, and P38 were evaluated via Western blotting in CS + LPS-induced mice lung tissue(n = 6). **C** H&E staining was performed on heart, liver, spleen, kidney, and brain tissues from CS + LPS-induced COPD mice (Scale bar = 200 μm). ^###^*P* < 0.001 vs control group; ^***^P < 0.001 vs CS + LPS group

**Fig. 7:**
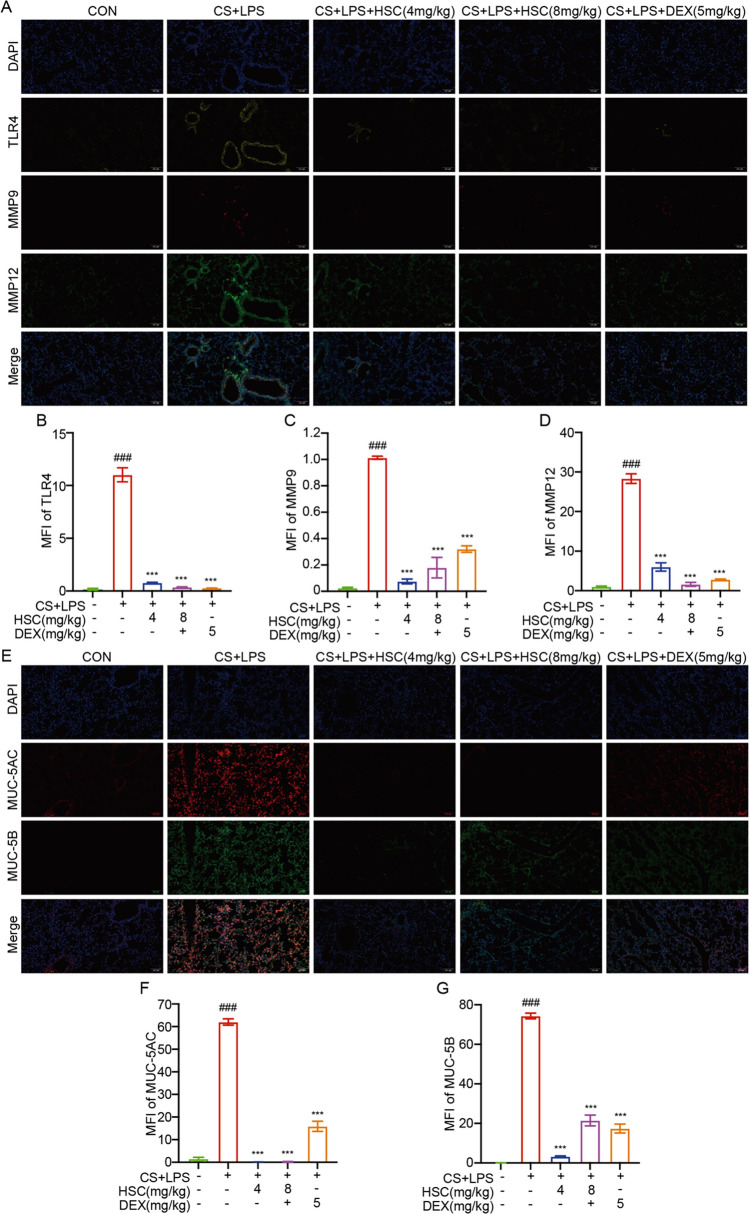
HSC alleviates COPD-related inflammation and airway remodeling via TLR4/NF-κB/MAPK pathway inhibition. **A**–**D** Multiplex immunofluorescence staining for TLR4, MMP9, and MMP12 in lung tissue of COPD mice and the statistical chart of MFI (Scale bar = 100 μm). **E**–**G** Multiplex immunofluorescence staining for MUC-5AC and MUC-5B in lung tissue of COPD mice and the statistical chart of MFI (Scale bar = 100 μm). ^###^*P* < 0.001 vs control group; ^***^P < 0.001 vs CS + LPS group

Histological analysis revealed that HSC significantly inhibited alveolar wall disorder, inflammatory cell infiltration, and hemorrhage induced by CS + LPS (Fig. 5K). HSC attenuated the elevated expression of TLR4, phosphorylated P65, IKKα/β, IκBα, JNK, ERK1/2, and P38 in COPD mice models (Fig. 6A, B). Furthermore, H&E staining of various organs indicated that HSC exhibited minimal toxicity (Fig. 6C). Multiplex immunofluorescence analysis highlighted that treatment with HSC markedly diminished the expression of TLR4, MMP9, MMP12, MUC-5AC, and MUC-5B proteins (Fig. 7A–G). In summary, HSC alleviated inflammatory symptoms in COPD mice by suppressing the NF-κB/MAPK signaling pathways overactivation and reducing the expression of smoking-associated factors, including MMP9, MMP12, MUC-5AC, and MUC-5B. These findings indicate that HSC shows potential as an effective therapeutic approach for mitigating COPD-related inflammation, excessive mucus production, and changes in airway structure.

## Discussion

The pathogenesis of COPD is multifactorial and involves an imbalance between proteolytic enzymes and their inhibitors, oxidative stress, and chronic inflammation [37]. COPD is marked by irreversible structural changes in the airways, including thickening of the airway wall and smooth muscle layer, increased mucus secretion, and alterations in epithelial cell morphology [38]. Consequently, COPD is often considered a mucus-obstructive disease, characterized by excessive mucus protein production [39]. The two main secreted airway mucins, MUC-5AC and MUC-5B, together form the mucus layer that lines the airways [40]. While MUC-5B is essential for maintaining airway homeostasis and antimicrobial defense, MUC-5AC plays a minimal role under normal conditions. However, during lung injury, MUC-5AC becomes a pathogenic glycoprotein [41]. In disease states, both MUC-5AC and MUC-5B levels are elevated, with a disproportionately higher increase in MUC-5AC [42]. In addition, COPD also manifests significant pathological changes in the small airways, accompanied by a progressive decline in lung function. Related studies have shown that in CS + LPS-stimulated mouse models, a significant increase in airway resistance and a significant decrease in airway flow rate were observed [12]. However, in this study, we have not yet systematically evaluated the lung function of mice, which will be one of the key directions for future research. Previous studies have shown that demethylation of the MUC-5B promoter region leads to increased gene expression [18]. In this study, we found that methylation at the chr11:1243469 position in the MUC-5B promoter region was significantly reduced following CSE + LPS exposure. Treatment with HSC effectively restored methylation levels at this site, suggesting that promoter methylation at chr11:1243469 position may play a critical role in the pathogenesis and treatment of COPD.

Matrix metalloproteinases (MMPs) are a group of structurally related metalloendopeptidases that are key mediators of extracellular matrix remodeling after tissue injury [43]. CS exposure contributes to COPD progression by disrupting the balance between MMPs and their natural inhibitors, tissue inhibitors of metalloproteinases (TIMPs), thereby impairing lung tissue repair [44]. MMP9 and MMP12 have been identified as potential biomarkers for COPD, as their levels are significantly increased in the airways of affected individuals and are considered promising targets for therapeutic intervention [45]. In this study, we observed that CSE + LPS exposure significantly increased the gene and protein expression of MMP9 and MMP12. Notably, treatment with HSC reversed these elevations, indicating its potential to attenuate airway remodeling and improve COPD-related pathological changes. At present, our research results have only revealed the regulatory role of HSC on MMPs at the transcriptional and translational levels. In subsequent experiments, we will assess the enzyme activity through gelatin zymography and further analyze the expression of TIMP proteins to more comprehensively elucidate the mechanism of protease-antiprotease imbalance. These contents constitute the core part of our future work plan.

Several cell types-including epithelial and endothelial cells, monocytes, macrophages, dendritic cells, T lymphocytes, and B lymphocytes-express TLRs. Among these, TLR4 specifically recognizes LPS from Gram-negative bacteria and plays a key role in infectious COPD exacerbations. In addition to bacterial LPS, exogenous oxidants and CS components can also activate TLR4 in the lungs [46].

TLR4 serves as an upstream regulator of the NF-κB signaling pathway through interaction with the adaptor protein MyD88. This interaction promotes the recruitment of interleukin-1 receptor-associated kinases (IRAKs) and TNF receptor-associated factor 6 (TRAF6), which in turn activate the IKK complex. This leads to phosphorylation, nuclear translocation of NF-κB, and the upregulation of pro-inflammatory cytokines such as TNF-α, IL-6, and IL-1β. These cytokines play essential roles in driving inflammation and are found at elevated levels in the lungs of COPD patients[47]. The MAPK signaling pathway also plays a crucial role in COPD progression by mediating cell chemotaxis, airway and alveolar remodeling, corticosteroid resistance, and airflow limitation [48]. TLR4 activation has been shown to simultaneously both the NF-κB and MAPK pathways [11]. In this study, molecular docking, CETSA, and SPR analysis confirmed that HSC binds directly to TLR4. Moreover, treatment with the TLR4 inhibitor TAK-242 suppressed the overactivation of the NF-κB and MAPK signaling pathways, indicating that HSC modulates these downstream pathways through TLR4 binding, thereby reducing inflammation and contributing to COPD treatment.

Oxidative stress is crucial in the inflammatory processes of COPD, with its primary characteristic being an excess of oxidative activity over the body’s antioxidant capabilities. Exposure to CS and environmental pollutants leads to elevated levels of ROS and reactive nitrogen species (RNS) in the lungs, thereby inducing airway inflammation and exacerbating the inflammatory response. Inflammatory cells that become activated produce additional ROS, creating a self-reinforcing cycle that substantially heightens oxidative stress. Within COPD, the disequilibrium between oxidant generation and antioxidant mechanisms may contribute to further disease progression [49]. Nevertheless, this study did not investigate whether HSC exhibit anti-inflammatory effects via oxidative damage pathways using markers related to oxidative damage, representing a limitation of the research and highlighting an area for future exploration.

In this study, HSC exhibits promising characteristics that support its potential for clinical translation in the treatment of COPD. Currently, inhaled corticosteroids such as budesonide and fluticasone are widely used to manage COPD inflammation. However, their long-term use is often limited by systemic side effects, increased risk of infections, and poor efficacy in patients with steroid-resistant inflammation [50–52]. In contrast, HSC, as a naturally derived triterpenoid saponin, may offer a safer and mechanistically distinct alternative or adjunctive therapeutic approach. Our MTT assays demonstrated that HSC had negligible cytotoxicity in vitro, and H&E staining of major organs in treated mice revealed no signs of tissue damage, supporting its favorable safety profile. Furthermore, HSC exerted potent anti-inflammatory effects in vivo, suggesting a therapeutic advantage over corticosteroids, particularly in scenarios where steroid responsiveness is compromised.

Pharmacokinetic studies have further indicated that HSC is druggable. Preclinical pharmacokinetic and pharmacodynamic studies demonstrate that HSC has favorable characteristics for clinical translation, including good bioanalytical stability, clear dose–effect correlation, and significant anti-inflammatory effects in ulcerative colitis models. The compound shows rapid absorption (*T*_max_ ~ 1 h), measurable exposure (*C*_max_ ~ 8.18 μg/mL), and effective cytokine suppression. Its PK/PD relationship fits an *E*_max_ model, supporting rational dose prediction [53]. These findings suggest HSC is a promising candidate for further clinical development, though human studies are needed to confirm efficacy and optimize dosing.

In addition, HSC has a history of use in traditional medicine formulations for treating respiratory tract inflammation and cough-related symptoms, with no major toxicity reported at therapeutic doses. This empirical background reinforces its safety and practical feasibility for further development. Taken together, these pharmacological and pharmacokinetic attributes support the potential of HSC as a novel therapeutic agent for COPD. Nonetheless, future research should include in vivo efficacy studies, comparative trials with standard-of-care drugs, and comprehensive ADME/toxicology profiling under GLP conditions to establish a solid foundation for clinical advancement.

## Conclusion

In summary, this study demonstrates that HSC can directly interact with TLR4, effectively suppressing the NF-κB/MAPK signaling pathways overactivation. This interaction reduces inflammation in COPD, restores protease-antiprotease balance, and mitigates excessive production of mucus. These findings highlight the potential of HSC as an effective therapeutic approach for managing COPD.

## Supplementary Information

Supplementary file 1.

Supplementary file 2.

Supplementary file 3.

## Data Availability

Requests for data access should be sent to the corresponding authors.

## Abbreviations

COPD: Chronic obstructive pulmonary disease
HSC: Hederasaponin C
LPS: Lipopolysaccharide
CSE: Cigarette smoke extract
CS: Cigarette smoke
CXCL-1: Chemokine (C-X-C motif) ligand 1
CXCL-8: Chemokine (C-X-C motif) ligand 8
TNF-α: Tumour Necrosis Factor alpha
CXCR-2: CXC chemokine receptor 2
IL-1β: Interleukin-1 beta
IL-6: Interleukin 6
ROS: Reactive oxygen species
TLRs: Toll-like receptors
TLR4: Toll-like receptor-4
NF-κB: Nuclear factor kappa B
MAPK: Mitogen-activated protein kinase
MMP9: Matrix metalloproteinase 9
MMP12: Matrix metalloproteinase 12
MMPs: Matrix metalloproteinases
TIMPs: Tissue inhibitors of metalloproteinases
MUC-5AC: Mucin-5AC
MCU-5B: Mucin-5B
PDE4: Phosphodiesterase-4
MyD88: Myeloid differentiation primary response gene 88
IRAKs: Interleukin-1 receptor-associated kinases
TRAF6: TNF receptor-associated factor 6
IKK: Inhibitor of kappa B kinase
TAK242: (R)-Ethyl6-(N-(2-chloro-4-fluorophenyl) sulfamoyl) cyclohex-1-enecarboxylate
CETSA: Cell thermal shift assay
SPR: Surface plasmon resonance
ELISA: Enzyme-linked immunosorbent assay
H&E: Hematoxylin and eosin
BALF: Bronchoalveolar lavage fluid
NLR: Neutrophil–lymphocyte ratio
PLR: Platelet-lymphocyte ratio
RCI: Red blood cell index
PLT: Platelet
RBC: Red blood cell
Hb: Hemoglobin
Lym: Lymphocyte
HDAC2: Histone deacetylase 2
qRT-PCR: Quantitative real-time PCR
MTT: 3-(4,5-Dimethyl-2-thiazolyl)-2,5-diphenyl-2-H-tetrazolium bromide
DEX: Dexamethasone
DMSO: Dimethyl sulfoxide
PMSF: Phenylmethylsulfonyl fluoride
IκBα: Inhibitor of kappa B α
JNK: C-Jun N-terminal kinase
ERK: Extracellular signal-regulated kinase
HRP: Horseradish peroxidase
RNS: Reactive nitrogen species
