# Supplementary figures and images for: Hederasaponin C ameliorates chronic obstructive pulmonary disease pathogenesis by targeting TLR4 to inhibit NF-κB/MAPK signaling pathways
Source: Chin Med. 2025 Jul 3;20:104. doi: 10.1186/s13020-025-01155-5 (PMC12225042; doi:10.1186/s13020-025-01155-5)

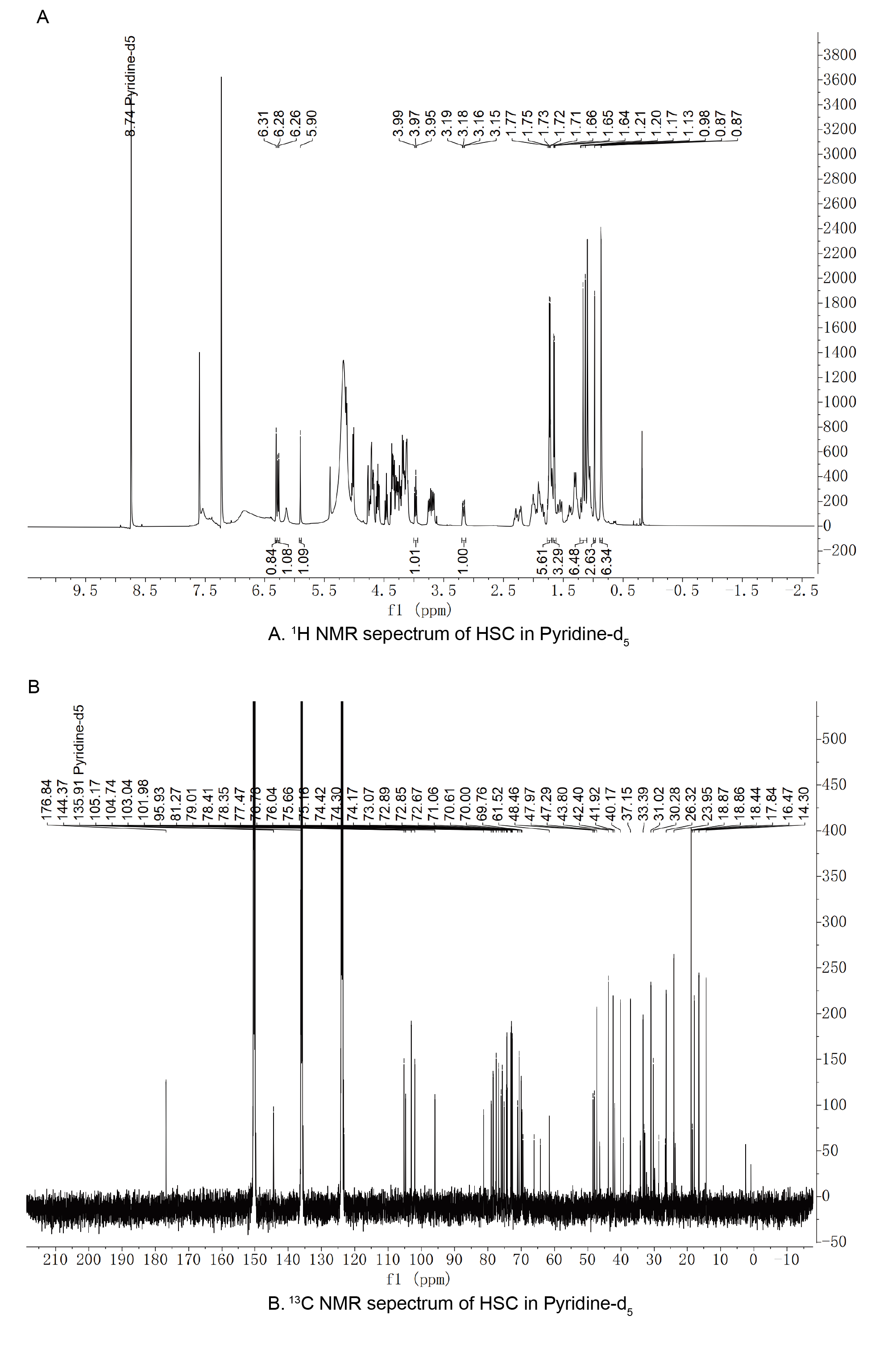

Supplement: Supplementary file 1 [file 13020_2025_1155_MOESM1_ESM.png]
